# Toe gaps and their assessment in footwear for people with diabetes: a narrative review

**DOI:** 10.1186/s13047-020-00439-3

**Published:** 2020-12-04

**Authors:** Petra Jones, Sicco A. Bus, Melanie J. Davies, Kamlesh Khunti, David Webb

**Affiliations:** 1 Leicester Diabetes Centre, Leicester General Hospital, University Hospitals of Leicester, Gwendolen Road, Leicester, LE5 4PW UK; 2 Diabetes Research Centre, University of Leicester, Leicester General Hospital, Leicester, UK; 3 Amsterdam UMC, University of Amsterdam, Rehabilitation Medicine, Amsterdam Movement Sciences, Amsterdam, the Netherlands; 4 NIHR Leicester Biomedical Research Centre, University of Leicester, Leicester, UK

**Keywords:** Diabetes, Foot, Ulcer, Fit, Footwear, Shoes, Measurement

## Abstract

**Background:**

Adequate footwear fit is critical in preventing diabetes-related foot ulcers. One important element is the toe gap, the difference between foot length and internal footwear length available to the foot. We summarised the literature on toe gaps in studies assessing footwear worn by people with diabetes, the methods used to measure both foot length and internal footwear length and identify ambiguities which may impact on toe gap assessment in clinical practice, and suggest pragmatic solutions.

**Methods:**

The Google Scholar database was searched to April 2020 for peer-reviewed studies using keywords related to incorrectly fitting or ill-fitting and diabetes, foot and ulcer which returned 979 results. Included studies within this narrative review encompassed toe gap measurement to assess footwear worn by people with diabetes.

**Results:**

A total of eight studies were included after full paper review. Toe gap ranges as used in assessments of footwear worn by people with diabetes vary, with a minimum of 1.0–1.6 cm and a maximum of 1.5–2.0 cm, as do methods of measuring internal footwear length. Only three published studies suggested possible measuring devices.

**Conclusions:**

Toe gap ranged as used when assessing footwear fit in people with diabetes vary and a gold standard device for internal footwear length measurement has yet to emerge. International guidelines provide welcome standardisation, but further research is needed to evaluate both the effect of toe gap ranges upon pressure, plantar stress response and ulceration and available measuring devices to facilitate development of toe gap measurement protocols that may further enhance consistency in practical assessments.

## Background

The cost of diabetes-related foot ulceration comprises almost 1 % of the National Health Service’s usual annual budget costing up to £1.13 billion per year [1] with 90% of these costs associated with ulcer care rather than prevention [2]. Around 2% of people with diabetes in the UK are likely to develop a foot ulcer each year [3]. In the UK, 3.9 million people have a diagnosis of diabetes [4] which suggests up to 78,000 people (2%) may potentially develop a foot ulcer each year. Lifetime prevalence of foot ulceration in people with diabetes may be as high as 30% [5]. Over three years of follow-up, risk of all-cause mortality associated with foot ulceration can be 22% higher than in those with diabetes who do not develop ulcers [6]. In those with severe ulcers, mortality can be as high as 14% within a year [7]. Besides increased risks of mortality [8] there is also an increased risk of lower limb amputation [9].

In 2015, NICE estimated that £5.4 million is spent on bespoke footwear in the UK: a figure which is likely now to be significantly higher [10]. Correctly fitting footwear is a key component in preventing diabetes-related foot ulceration (DFU) by reducing inflammation and callus development. Incorrectly fitting footwear is any footwear that is too short or long, too narrow or wide or which impairs normal function of the foot to adversely affect gait [11]. ‘Footwear fit’ refers to adequacy of footwear length, width and height relative to the size of the foot. The prevalence of ulcers which are self-reported through interview or survey as caused by new shoes, non-specific rubbing by inflexible or abrasive footwear materials, and incorrect fit range between 19.1 and 54.4% [12–15] (Supplemental Table 1). The precise number of ulcers caused by inappropriate fit as distinct from footwear material or condition is unknown but a landmark study by Armstrong et al. (*n* = 360) found 13% of ulcers occurred on the dorsum of digits, 40% at the hallux and a further 10% at the plantar aspect of digits, likely locations influenced by footwear abrasion and therefore fit [16].

One aspect of footwear fit is toe gap. Toe gap is the difference between foot length (the distance from heel to the apex of the longest toe [17]) and the internal footwear length. The internal footwear length is the linear distance available to the foot from the interior of the shoe’s heel counter to the interior toe box. The shape of the toe box, and the associated volume of space available to the foot, is known to affect the in-shoe pressures of healthy feet [18]. Insufficient toe gap may also compromise toe position. Footwear length is also a significant feature of footwear comfort [19]. However, there is much we don’t know about toe gap. The aim of this review is to summarise the ranges of toe gaps applied in the assessment of footwear worn by people with diabetes as part of ulcer prevention, the methods used to measure both foot and internal footwear length and to discuss ambiguities which may impact on toe gap assessment in clinical practice.

## Methods

In order to examine both the guidelines and literature surrounding footwear fit for people with diabetes, we carried out a Google Scholar database search (any date until 15 April 2020). The search string ((“incorrectly fitting” OR “incorrectly-fitting” OR “ill-fitting” OR “ill fitting”) AND (“shoes” OR “footwear”) AND (“diabetes”) AND “foot” AND “ulcer”) returned 979 results excluding patents and citations. The search criteria do not directly refer to “toe gap” given the variety of names used to describe this measurement, for instance “range corresponding to shoe size” or “space between top of longest toe and shoebox”. Only peer-reviewed studies including people with diabetes were included. Eligibility criteria included any study (i) involving people with diabetes that (ii) attempted to apply a quantitatively defined toe gap as part of footwear assessment. Studies were assessed by title and abstract to determine eligibility from which 15 studies were selected with 10 excluded after full paper review. A further 12 full papers were identified through reference analysis of which 3 were included, totalling 8 relevant studies (Table 1) [20–27] which formed the basis of this narrative review. Reasons for exclusion included limited discussion of quantifiable physiological fit (*n* = 9), discussion of fit only in healthy participants (*n* = 1), limited numbers of people with diabetes or unknown whether any have diabetes (*n* = 2), no discussion of toe gaps, only footwear size (*n* = 5) or where the focus was on foot change and morphology rather than incorrect fit (n = 2).

**Table 1:** Studies assessing toe gap in footwear worn by people with diabetes

Study	Fit Criteria Used					Heel (cm)	Toe Gap (cm)		Type / No. Participants					Gender	Footwear Type
	T	L	B	M	F		Min	Max	P	CG	D	DN	Age	M / F	
Barwick [20]	✓	✓	–	✓	✓	2	1.0	2.0	555	–	12	159	–	–	COMMERCIAL & BESPOKE
Chantelau [21]	–	–	–	–	✓	–	1.0	1.5	–	100	–	568	64	–	COMMERCIAL
Chicharro-Luna [22]	✓	✓	✓	✓	✓	3.0	1.0	1.5	–	–	91	17	59.6 ± 10.3 (DN) 57.7 ± 13.7 (D)	13/4 (DN) 57/34 (D)	COMMERCIAL
Fan [23]	✓	✓	✓	✓	✓	2.5	1.3	–	–	–	–	56	55.8 ± 13.2	26/30	COMMERCIAL
Isip [24]	✓	–	–	–	✓	–	1.0	2.0	–	–	170	–	64	46/124	COMMERCIAL
Litzelman [25]	–	–	–	–	✓	–	1.9	–	–	–	352		60.4 ± 9.6	–	COMMERCIAL & BESPOKE
McInnes [26]	–	–	–	–	✓	–	1.0	1.5	–	118	–	82	40–75	–	COMMERCIAL
Nancarrow [27]	✓	✓	✓	✓	✓	2.5	1.0	–	–	–	87	13	–	54/46	COMMERCIAL & BESPOKE

Fit Criteria Used: T = Footwear type, L = Fastening type e.g. laces, B = Toe box shape, M = Material type, F = Fit. Heel = Maximum heel height (cm)

Type / No. of Participants: P (Patient group without diabetes); CG (Control group i.e. without diabetes); D = with diabetes; DN = with both diabetes and neuropathy

Age: Usually mean with standard deviation shown where available. Where unavailable, an age range is provided

## Discussion of toe gap within footwear assessments

### 1.0–1.5 cm toe gap range

Chantelau and Gede carried out an anthropometric assessment of foot length and width characteristics of people with diabetes as compared to those without [21] adopting a toe gap range (1.0–1.5 cm) used by the German Shoe institute in children’s shoes (Das Mass-System fur Kinderschuhe, Richtlinien des ‘Arbetskreises Kinderschuhe’ WMS standard 1990, since amended to 0.9–1.5 cm) applied to adults with a mean age of 64 years. Whilst the feet of those with diabetes were found to be broader than shoe industry reference data used in available sizes, foot length was said to match well with available commercial footwear.

This benchmark 1.0–1.5 cm toe gap range has been applied in other studies including McInnes et al. [26] where toe gap differences were assessed in people with diabetes and neuropathy (*n* = 85) and those without diabetes (*n* = 118) to explore the hypothesis that people with diabetes wear shoes that are too small to increase the sensation of fit. The mean toe gap was not significantly different between groups – a large proportion of both groups in fact were wearing footwear outside this 1.0–1.5 cm toe gap range (82% within the diabetes and neuropathy group and 66% among those without diabetes) [26]. This study is unique in describing the range and distribution of toe gaps found within footwear worn by people with diabetes during daily living. The toe gap ranged from 0 (i.e. no gap at all) to a maximum of 3.7 cm with a reported median of 1.2 cm, where 47% of those with diabetes wore footwear that was too long (> 1.5 cm toe gap) and 35% too short (a 0.9 cm or less toe gap). The 1.0–1.5 cm toe gap was applied in McInnes [26] with an acknowledgement that this range is not evidence based but applied on the basis of past use by others.

Chicharro-Luna et al. [22] also used this 1.0–1.5 cm toe gap to assess footwear length within a convenience sample of 108 people with diabetes, 17 of which had neuropathy, and all of which had a diabetes duration of more than five years. Among the 160 items of closed footwear (as opposed to open toe sandals) only 65 (40.6%) were of correct length, with 63 (39.4%) wearing footwear that was too large (> 1.5 cm toe gap) and 32 (20%) wearing footwear that was too small (< 1.0 cm toe gap).

#### A literal rule of thumb

The minimum toe gap applied to assess footwear worn by people with diabetes is typically 1 cm (*n* = 6) [20–22, 24, 26, 27] but in some instances a more generous rule of thumb is applied [23, 25] presumably intended as an approximate measure. In Litzelman [25] 352 patients were included in evaluation of footwear characteristics as predictors of DFU, of whom 63 ulcerated. The evaluation of footwear characteristics included a toe gap assessment that was based on the healthcare professional’s three-quarter inch (1.9 cm) thumb (too short if less than this, and too long if greater). No statistically significant relationship was found between appropriate footwear length and ulceration. Similarly, toe gap assessment was based on a thumbnail’s length (half inch or 1.3 cm) in Fan et al. [23] as part of the evaluation of an educational and self-care intervention to improve footwear choice (style, laces/fastening, materials and fit), which led to a reduction in the proportion of people with diabetes (*n*=56) wearing incorrectly fitting shoes at 3-month follow-up. (19.6% at baseline to 7.1% at follow-up).

### 1.0–2.0 cm toe gap range

The updated Guidelines on the Prevention and Management of Diabetic Foot Disease from the International Working Group on Diabetic Foot (IWGDF) [28] advise that people with diabetes but no loss of protective sensation (LOPS) or peripheral artery disease (PAD) should select properly fitting off-the-shelf footwear, recommending a toe gap between 1 and 2 cm. Those with diabetes and LOPS and/or PAD are advised to take “additional care” with selecting and fitting. This recommended toe gap is used in Isip [24], an assessment of footwear worn by 170 Filipino patients with diabetes. After exclusion of those wearing inappropriate footwear e.g. flipflops, sandals and open toed footwear, toe gap was assessed in 78 patients of whom 34 (21 female, 13 male) wore footwear of incorrect length (43.6%). The IWGDF toe gap range of 1-2 cm was also applied in Barwick [20] a study which included 171 diabetes inpatients and investigated the prevalence (49%) and factors associated with wearing inadequate outdoor footwear (including Velcro or laces, appropriate heal height, and other factors as well as fit). However, Barwick et al. [20] did not collect information on fit directly (relying on a patient questionnaire) which was a limitation of this study.

#### Toe gap fit and diabetes-related ulceration

We were unable to find any studies which specifically evaluated these toe gap ranges in the most frequently worn footwear to assess whether there is any correlation with those who did or did not ulcerate. We were also unable to find studies which evaluate toe gaps in relation to in-shoe plantar pressures. It seems reasonable to suggest that a shorter toe gap than 1 cm might significantly increase the risk of pressure-related sores on the apex of the longest toe from cramping of the toes, but we were unable to locate any study to this effect. Similarly, there is an absence of studies to evaluate whether a toe gap longer than 1.5 or 2.0 cm causes friction-related abrasions from increased movement.

How much toe gap is biomechanically necessary for walking and when does mechanical stress begin to become excessive and lead to calluses, blisters or friction? It seems reasonable to suggest that insufficient toe gap might increase pressure at the toes and might increase friction and shear associated with increased skin temperatures (sometimes referred to as ‘plantar stress response’) [29]. Monitoring insoles capable of analysing vertical in-shoe pressures have been around for many years but have yet to be used to analyse pressure patterns associated with varying toe gaps. This may be due to the specialist skillset required to calibrate and utilise in-shoe pressure monitoring insoles, as well as their cost. There may also be an issue around skill mixing for healthcare professionals in terms of whether the assessment of pressure, toe gap and foot size is seen as part of an orthotist/shoe-technician, biomechanical role or as part of podiatry.

Some individuals with diabetes may also have rheumatoid arthritis. Rheumatoid arthritis also has guidelines which specify recommended toe gap (e.g. 0.6–1.1 cm [30] or a minimum 1.0–1.3 cm [31]). This may lead to conflict for patients with both conditions. Again, this suggests there is some value in real time or habitual analysis of in-shoe forefoot plantar pressures associated with various toe gap ranges thereby providing evidence supporting the toe gaps applied.

Real-time free-living pressure data immediately prior to inflammation, injury or ulceration is preferable to snapshots within laboratory-based conditions.

#### Toe gap measurement

While our understanding is incomplete, a more pressing practical concern is how to measure toe gap. Currently, the IWGDF guidelines do not specify the most appropriate methodology for measuring either the foot or the more difficult to access internal footwear length within a shoe. In the 8 studies of shoe fit we found that of those focused on people with diabetes, four employed a Brannock or similar device (Table 2), using slide rules to position and measure the foot [21, 22, 24, 26] (as recommended by the DFAGF [33]) to obtain this distance from the heel to longest toe. In two studies a rather literal rule of thumb was used to estimate toe gap presumably by pressing on the toes [23, 25].

**Table 2:** Toe gap fitting standards applied to footwear of people with diabetes

**Study, Year**	**Type of Study**	**Toe Gap (cm)**		**Toe Gap Justification**	**Fit Measurement Method**		
		**Min**	**Max**		**Measure Position**	**Foot**	**Footwear**
Barwick, 2019^a^ [20]	Cohort study	1.0	2.0	IWGDF and Diabetic Foot Australia guidelines cited	–	–	N/A
Chantelau, 2002^b^ [21]	Case-control	1.0	1.5	Gap used by German Shoe institute in children’s’ shoes (WMS standard 1990) now 0.9–1.5 cm	STANDING	WMS	N/A
Chicharro-Luna, 2020 [22]	Cohort study	1.0	1.5	Based on guidance within an article by Edelstein [32]	STANDING	BRANNOCK	CEGI DEVICE
Fan, 2014 [23]	Cohort study	1.3	–	Thumbnail’s length, half inch. Unattributed.	–	–	–
Isip, 2016 [24]	Cohort study	1.0	2.0	IWGDF guidelines cited (43.6% wearing footwear of incorrect length based on 78 measured)	STANDING	BRANNOCK	PLUS 12 MED
Litzelman, 1997 [25]	Cohort study	1.9	–	Based on nurse-clinician’s thumb width of 3/4 in.	STANDING	THUMB	–
McInnes, 2012 [26]	Case-control	1.0	1.5	Chantelau recommendations cited [21]	STANDING	BRANNOCK	ISSG
Nancarrow, 1999 [27]	Cohort study	1.0	–	Approx. 1 cm on weight bearing. Unattributed.	STANDING	SELF ASSESSMENT	
**Guideline**	**Type of Study**	**Min**	**Max**	**Toe Gap Justification**	**Measure Position**	**Foot**	**Footwear**
IWGDF, 2019 [28]	N/A	1.0	2.0	Unattributed.	STANDING	–	–
Diabetic Foot Australia, 2018 [33]	N/A	1.0	2.0	Unattributed.	STANDING	BRANNOCK	BRANNOCK

% Incorrectly fitted: *N/A* Not assessed. – No data available

Measurement Method: *ISSG* Internal Shoe Size Gauge, *CEGI device* Combined foot measurement device and plastic internal footwear length gauge

^a^ Barwick did not collect information on fit directly (rather through a survey) which is a limitation of the study

^b^ Chantelau evaluated foot anthropometrics in relation to available industrial footwear size

Calculation of toe gap necessitates advice on how to obtain the internal footwear length. The IWGDF guidelines [28] do not discuss this (Table 2). The DFAGF recommends using a Brannock device [33] to obtain the outer footwear dimensions but this relies on an estimate of hidden material thickness at the toe to make an informed guess regarding the available internal length within a shoe. Only three studies [22, 24, 26] attempted an actual measurement of the internal shoe length available for the foot (Table 2). In Isip et al. [24] a flexible Plus12med device was utilised. This is an L-shaped device which is placed inside the shoe resting against the heel using a stiff extendable measurer to reach the internal front end of the footwear. An alternative method was used by McInnes et al. [26] involving a SATRA internal shoe size gauge. This is a metal device which fits inside the shoe and extends outwards until it reaches the internal front end of the footwear. In this instance, it was adapted to also measure internal footwear length rather than shoe size but a device specifically for internal foot length measurement is now available (SATRA STD 225 M). Finally, in Chicharro-Luna et al. [22], a CEGI instrument was used which is capable of measuring both the foot and the internal footwear length using a plastic gauge on the side of the device.

Some space within a shoe may not be accessible to the toes and this should not be included in any measurements. The tip of the Plus12med device is 1.1 cm in height thereby excluding some unusable space. Recent innovations also include independent footwear research and testing organisation SATRA’s innovation in-shoe gauge, a foot shaped device similar to a shoe stretcher which extends inside the shoe until it occupies and thereby measures the usable internal length within footwear (STD 225E). This is an interesting new measuring tool although it is limited to assessing toe gap for a particular shoe size, with the device’s width based on the average rather than individual joint girth and it is currently not commercially available. Given the absence of a gold standard measure, there is a need for studies evaluating these and other potential tools [34] and comparing their accuracy and ease of use within podiatry and orthotics. This will enable further development of guidance around how practically internal shoe space should be measured when assessing toe gap in off-the-shelf and custom-made footwear, with the goal of reducing calluses, friction or ulceration.

Another area requiring further guidance is ensuring adequate toe gap in feet of unequal lengths. Feet of unequal length are common. A study measuring the foot length and ball width of 6800 randomly selected individuals within shoe shops by skilled fitters found that feet were of equal length in only 33% of cases [35]. Study limitations included the absence of both demographic information (e.g. BMI, proportion with diabetes) and set procedures (two rulers were used for foot length measurement rather than the more conventional Brannock device). A further study found a 25% prevalence of feet with unequal length (defined here as more than a 0.5 cm difference) based on sliding calliper measurement of the feet of people with diabetes (*n* = 111) [36].

Most studies which assess the toe gap by reference to the longest toe (typically the hallux but sometimes the second toe) do not specifically address this question: what to do when a pair of shoes is incorrectly fitted because only one shoe contains the necessary toe gap. The generous 1 to 2 cm range should encompass some of these differences but at what stage would the expense of made-to-measure shoes be justified for someone without LOPS or PAD as a precaution against foot trauma or friction due to ill-fitting shoes from feet with asymmetrical length?

#### Dynamic foot shape change

Another question is whether the majority of foot shape change is incurred during the transition from sitting to standing or whether maximal foot length occurs during movement? It is important to determine how much stretch and flexibility is required by forefoot material to encompass dynamic changes in foot length during walking and other daily living activities. There is some evidence that foot volume while a person is seated may change by 2% even after 10 min of walking [37], although this study is limited to a small number of healthy participants rather than people with diabetes. This raises an interesting question of whether foot length alters any further during walking, and if assessment during standing is sufficient for toe gap measurement? Few studies have been carried out due to the limitations of current technology in capturing foot morphology during walking. However, in a motion camera analysis of healthy participants (*n* = 34), the foot appears to lengthen after the toe strike phase by up to a mean 0.58 cm (SD 1.9) and then shortens by up to a mean 0.54 cm (SD 2.4) after heel off and prior to the toe pushing off [38]. At faster walking speeds this lengthening of the foot is reduced (0.58 ➔ 0.50 cm, *P* < 0.01). Similar studies are needed with both larger numbers of participants and specifically involving people with diabetes and different levels of neuropathy to further improve our understanding of biomechanically-required toe gaps and whether these are affected by body weight, aging and other factors. Most 3D foot scanning systems are too slow to capture dynamic changes in foot shape [39] and alternative high-speed cameras and structured light patterns or fluoroscopy within shoes too costly to set up, often necessitating both construction of raised walkways and specialist knowledge [40]. A technological solution is therefore required which is both cheap and easy to implement.

## Conclusions

Toe gap is deemed to be an important measure for appropriate fit of footwear yet there is no consensus as to how it should be measured or what the impact of toe gaps that are too small or too large is likely to be. There is an absence of consensus within these studies regarding minimum and maximum toe gaps, and a lack of objective evidence (in-shoe pressure, plantar stress response i.e. elevated temperatures, ulceration rates or days spent in remission) associated with them. The IWGDF guidelines are a welcome move towards standardisation with the recommended toe gap range of 1–2 cm with which healthcare practitioners and podiatrists can assess footwear worn by people with diabetes at risk of ulceration. However, further research is needed to evaluate the effect of toe gap ranges in standardised footwear upon ulceration rates and days spent in remission, or to investigate their relationship with pressure and plantar stress response. Promising new devices for measuring internal footwear length have recently emerged which have yet to be formally evaluated within clinical comparative studies. These could provide a much-needed gold standard tool for objective measurement of toe gap.

This will facilitate further development of the IWGDF guidelines to include a protocol or recommendations as to how toe gap should be measured to standardise measurement in footwear evaluation. The IWGDF guidelines are a powerful and above all living and evolving tool for healthcare practitioners which we hope will come to include protocols for footwear measurement to facilitate practical assessments.

## Supplementary Information

**Additional file 1: Supplemental Table 1.** Diabetes-related ulceration linked to footwear. **Supplemental Table 2.** Proportion of incorrectly fitting footwear worn by people with diabetes.

## Data Availability

Please contact author for data requests (systematic review data).

## Abbreviations

BMI: Body Mass Index
CI: Confidence interval
cm: Centimetres
DFAGF: Diabetic Foot Australia Guideline on Footwear
IWGDF: International Working Group on Diabetic Foot
LOPS: Loss of Protective Sensation
NICE: National Institute of Health and Care Excellence
DFU: Diabetes-related foot ulceration
PAD: Peripheral Artery Disease
SD: Standard Deviation
