# Supplementary material for: Toe gaps and their assessment in footwear for people with diabetes: a narrative review
Source: J Foot Ankle Res. 2020 Dec 4;13:70. doi: 10.1186/s13047-020-00439-3 (PMC7718668; doi:10.1186/s13047-020-00439-3)
Supplement: Supplementary file 1 — Additional file 1: Supplemental Table 1. Diabetes-related ulceration linked to footwear. Supplemental Table 2. Proportion of incorrectly fitting footwear worn by people with diabetes. [file 13047_2020_439_MOESM1_ESM.docx]

**SUPPLEMENTAL TABLE 1. Diabetes-related ulceration** **linked to** **footwear**

| **Paper** | **Year** | **Sample Size** | **Cohort** | **Ulcers**  **Linked to**  **Footwear (%)** | **Avg. Age**  **[SD] (years)** | **M / F**  **(%)** | **Type 2 / 1**  **(%)** | **Duration of Diabetes**  **(years)** |
| --- | --- | --- | --- | --- | --- | --- | --- | --- |
| Algarni [12] | 2013 | 209 | DFU | 19.1% | 59.0 [±14.8] | 79.9% / 21.1% | -- | 16.9 [±8.6] |
| Macfarlane [13] | 1997 | 669 | DFU | 20.6% | -- | -- | -- | -- |
| McGill [14] | 2005 | 30 | DFU | 54.4% | 59.0 | 74.4% / 25.6% | 92.8% / 7.2% | 10.3 |
| Premkumar [15] | 2017 | 301 | DFU(1st) | 21.9% | -- | 71.2% / 28.8%* | 86.4% / 13.6%* | -- |

**Key**

DFU = People with diabetes and a foot ulcer, DFU(1st) = People with diabetes and their first foot ulcer. *Breakdown only available for those whose ulcer was attributed to footwear.

**SUPPLEMENTAL TABLE 2. PROPORTION OF INCORRECTLY FITTING FOOTWEAR WORN BY PEOPLE WITH DIABETES**

|  | **Footwear Outcome** | **Fit Criteria Used** | | | | | **Toe Gap (cm)** | |
| --- | --- | --- | --- | --- | --- | --- | --- | --- |
| **Study** |  | **T** | **L** | **B** | **M** | **F** | **Min** | **Max** |
| Barwick [20] | 49.0% inadequate outdoor footwear | 🗸 | 🗸 | -- | 🗸 | 🗸 | 1.0 | 2.0 |
| Chantelau [21] | 65.9% incorrect width^1^ | -- | -- | -- | -- | 🗸 | 1.0 | 1.5 |
| Chicharro-Luna [22] | 66.7% incorrect length (DN)  58.1% incorrect length (D)^2^  95 / 160 closed footwear items (59.4%) incorrect length  63 / 160 (39.4%) footwear too large (> 1.5 cm toe gap)  32 / 160 (20%) footwear too small (< 1.0 cm toe gap) | -- | -- | -- | -- | 🗸 | 1.0 | 1.5 |
| Fan [23] | 19.6% incorrect length and width at baseline | 🗸 | 🗸 | 🗸 | 🗸 | 🗸 | 1.3 | -- |
| Isip [24] | 43.6% incorrect length | 🗸 | -- | -- | -- | 🗸 | 1.0 | 2.0 |
| Litzelman [25] | 30.0% incorrect length - wound on both feet  28.8% incorrect length - wound on one foot  41.9% incorrect length – no wounds  (Results not statistically significant, 0.60 K-value) ^3^ | -- | -- | -- | -- | 🗸 | 1.9 | -- |
| McInnes [26] | 82.0% incorrect length (number of participants) | -- | -- | -- | -- | 🗸 | 1.0 | 1.5 |
| Nancarrow [27] | 35% inadequate footwear (shoe causes injury to feet) | 🗸 | 🗸 | 🗸 | 🗸 | 🗸 | 1.0 | -- |

**KEY**

^1^ Based on mean average of percentage of feet broader than shoe width for male/female in size 7/8 (Table 1 results in Chantelau).

^2^ Proportion of feet. Based on c29.2% (<1 cm toe gap) + 37.5 (> 1.5 cm toe gap) = 66.7% incorrect length in feet of people with diabetes and neuropathy (DN).

Based on 18.4% (<1 cm toe gap) + 39.7% (> 1.5 cm toe gap) = 58.1% incorrect length in feet of people with diabetes without neuropathy (D).

^3^ Based on 30.0% (footwear too short) + 0% (footwear too long) = 30.0% (wound on both feet); 26.4% (footwear too short) + 1.9% (footwear too long) = 28.8%.

40.2% (footwear too short) + 1.7% (footwear too long) = 41.9% (Table 2 results in Litzelman).
